# Advancing Professionalism Through a Patient Safety and Quality Improvement Workshop in Radiation Oncology

**DOI:** 10.7759/cureus.89445

**Published:** 2025-08-05

**Authors:** Leslie Chang, Julia M Kim, Samantha I Pitts, Aditya Halthore, Khinh R Voong, Jean L Wright

**Affiliations:** 1 Department of Radiation Oncology, University of Minnesota, Minneapolis, USA; 2 Department of Radiation Oncology and Molecular Radiation Sciences, Johns Hopkins University, Baltimore, USA; 3 Department of Pediatrics, Johns Hopkins University School of Medicine, Baltimore, USA; 4 Armstrong Institute for Patient Safety and Quality, Johns Hopkins University, Baltimore, USA; 5 Department of Medicine, Johns Hopkins University School of Medicine, Baltimore, USA; 6 Department of Radiation Oncology, University of North Carolina, Chapel Hill, USA

**Keywords:** educational curricula, patient’s safety, professionalism education, quality improvement (qi), systems based practice, teaching and learning strategies

## Abstract

Background: Integrating a practice-based curriculum to cover essential aspects of patient safety and quality improvement (PSQI) in the outpatient setting can improve resident understanding and engagement in future projects within a radiation oncology residency program.

Objectives: To develop and pilot a workshop-based PSQI curriculum to lead residents through a simulated departmental quality improvement project and enhance understanding regarding tools and metrics used within a PSQI framework.

Methods: This pilot project was completed in the 2023-2024 academic year, with 13 residents in one radiation oncology program. This educational program included four two-hour interactive PSQI workshops. Residents applied PSQI tools to a simulated scenario that reflected a department priority: to improve time from computed-tomography simulation to adaptive radiation therapy. Residents completed a 5-point Likert survey and an institutionally developed PSQI in-training exam before and after receiving the program to evaluate understanding of key concepts, measurement methodologies, strategies for improvement, and attitudes towards the adoption of PSQI in clinical practice.

Results: The pilot workshop was successfully implemented during resident didactics. Although all thirteen residents were able to participate, eight (62%) residents completed all questionnaires. The curriculum led to improved comfort levels in applying PSQI techniques such as fishbone diagrams (pre-post mean Likert scores: 1.7 to 3.7, p< 0.01). PSQI in-training test scores significantly increased by 13% (57% to 70%, p<0.01).

Conclusions: The development and implementation of a PSQI curriculum in radiation oncology was feasible, improved baseline knowledge of PSQI techniques, and enhanced participant self-reported preparedness and skills for future PSQI projects.

## Introduction

Patient safety and quality improvement (PSQI) education is considered a priority within the Accreditation Council for Graduate Medical Education (ACGME). One of the common program goals includes residents systematically using quality improvement methods and implementation of changes with the goal of practice improvement [1]. In addition, ACGME systems-based practice milestones for radiation oncology residents includes demonstrating the knowledge and skills to develop, implement and analyze a quality improvement project [2]. PSQI education is also an important aspect of preparation for radiation oncology qualifying board examinations as safety, quality assurance and radiation protection comprise approximately 10% of the medical physics examination [3].

However, patient safety and quality management was identified as one of the biggest gaps in radiation oncology graduate medical education. Prior survey results indicated that only 34% of radiation oncology residents felt they had adequate training in patient safety and quality management [4]. Additionally, there are few ACGME-accredited radiation oncology training programs that have a formal PSQI education curriculum. Key barriers to effective PSQI education include lack of qualified faculty, lack of integration with training and lack of time [5]. Currently, there are few resources to assist in resident education in these topics and available references in patient safety and quality improvement are typically non-interactive, online curriculums or textbooks [6-8]. However, practice-based learning has shown trainees had improved understanding of PSQI concepts [4,9]. Integration of these concepts within a simulated project in the radiation oncology clinic during regularly scheduled didactics eliminates common barriers such as the lack of integration of PSQI education with training and the time to obtain data/metrics for evaluation of project efficacy. Interactive workshops in PSQI with basic principles allow trainees to obtain a greater comfort level in their ability to lead clinical safety programs and implement quality improvement (QI) projects [9]. Increased resident participation in QI is associated with the identification of projects that pertains to daily operations such as those centered on implementation of clinical practice guidelines or patient safety [10]. Additionally, active engagement in the improvement of clinical systems reduces frustration and clinician burnout [11].

Here we report the development and implementation of a workshop-based PSQI curriculum that leads residents through a simulated departmental quality improvement project in radiation oncology. Simulation has been successfully used in graduate medical education as an effective method of increasing understanding and interest in quality improvement and patient safety [12]. Enhancing resident understanding of PSQI methodologies can be adapted to real-time decision-making in the radiation oncology clinic.

Portions of this study were previously presented at the American Board of Medical Specialties Conference, Sept 24-26th, Chicago, IL, USA.

## Materials and methods

Setting and participants

We conducted a prospective, nonrandomized, observational study evaluating the effectiveness of a PSQI curriculum and simulated QI project involving 13 residents enrolled in the radiation oncology residency training program at Johns Hopkins University. Institutional Review Board determined the project was exempt research and approval was obtained for this project (IRB00395321). Participation in the study was voluntary. Informed consent was obtained from all participants prior to their inclusion in the study. Radiation oncology residency training is performed in an apprenticeship model from Postgraduate Year (PGY) 2 through PGY5. All residents were expected to attend the biweekly scheduled didactic curriculum. The project leadership included the chief resident and the vice chair of patient safety and quality improvement in radiation oncology. In addition, faculty from the Armstrong Institute for Patient Safety and Quality at Johns Hopkins University served in advisory roles.

Program description

The PSQI curriculum consisted of four two-hour interactive workshops with a resident and attending educator. The key topics included: 1) Project definition and Stakeholder engagement, 2) Measuring and Mapping, 3) Identifying and Implementing Interventions, 4) Measuring and Sustaining Improvement. Key goals for the curricula were completing the initial steps of a quality improvement project. This includes the definition of a SMART (specific, measurable, achievable, relevant, time-bound) aim, Stakeholder mapping, Swim Lane process mapping, Fishbone diagram creation, using a PDSA cycle and depicting QI data using a run vs control chart. Our curriculum was adapted from a resident curriculum developed through the Armstrong Institute for Patient Safety and Quality and the Hopkins Graduate Medical Education office. The base curriculum was developed with the intention of being tailored for specific use by different residency specialties. The lectures reviewed the Model for Improvement as well as the Lean Sigma DMAIC frameworks for defining, measuring, analyzing, improving and evaluating sustainability for a quality improvement project. Specific to radiation oncology, the sessions also reviewed patient safety and treatment quality assurance. In addition, the curriculum incorporated an interactive simulated QI project to improve communication during adaptive radiation planning during each workshop. The pre-defined aim was to improve time in days from computed tomography simulation to adaptive radiation therapy.

Data collection

A questionnaire was administered to residents before the introduction of the PSQI curriculum and at the end of the last session. This survey assessed residents' baseline knowledge and confidence in QI skills, including their understanding of key concepts, as well as perceptions towards QI using a five-point Likert scale questionnaire based on the validated Beliefs, Attitudes, Skills and Confidence in Quality Improvement (BASiC-QI) scale (Supplementary Material 1) [13]. Additionally, a 20-question exam extracting relevant PQSI questions regarding radiation oncology quality improvement as well as Institute for Healthcare Improvement [14] and AMA Health Systems Science modules [15] was administered.

Analysis

Descriptive statistics were used to summarize the quantitative data obtained from the pre-and post-implementation surveys. The distribution of paired difference scores was assessed using the Shapiro-Wilk test and found to significantly deviate from normality (p < 0.001). Therefore, a non-parametric Wilcoxon signed-rank test was used employed to assess significant individual differences in residents' understanding of quality improvement metrics before and after the curriculum implementation. P-values where p<0.05 were considered significant. Statistical data was analyzed using Stata 19 (StataCorp, College Station, TX, USA). Open-ended discussion and survey questions regarding participants' perceptions of PSQI and prediction of future clinical practice changes were captured.

## Results

**Figure 1:**
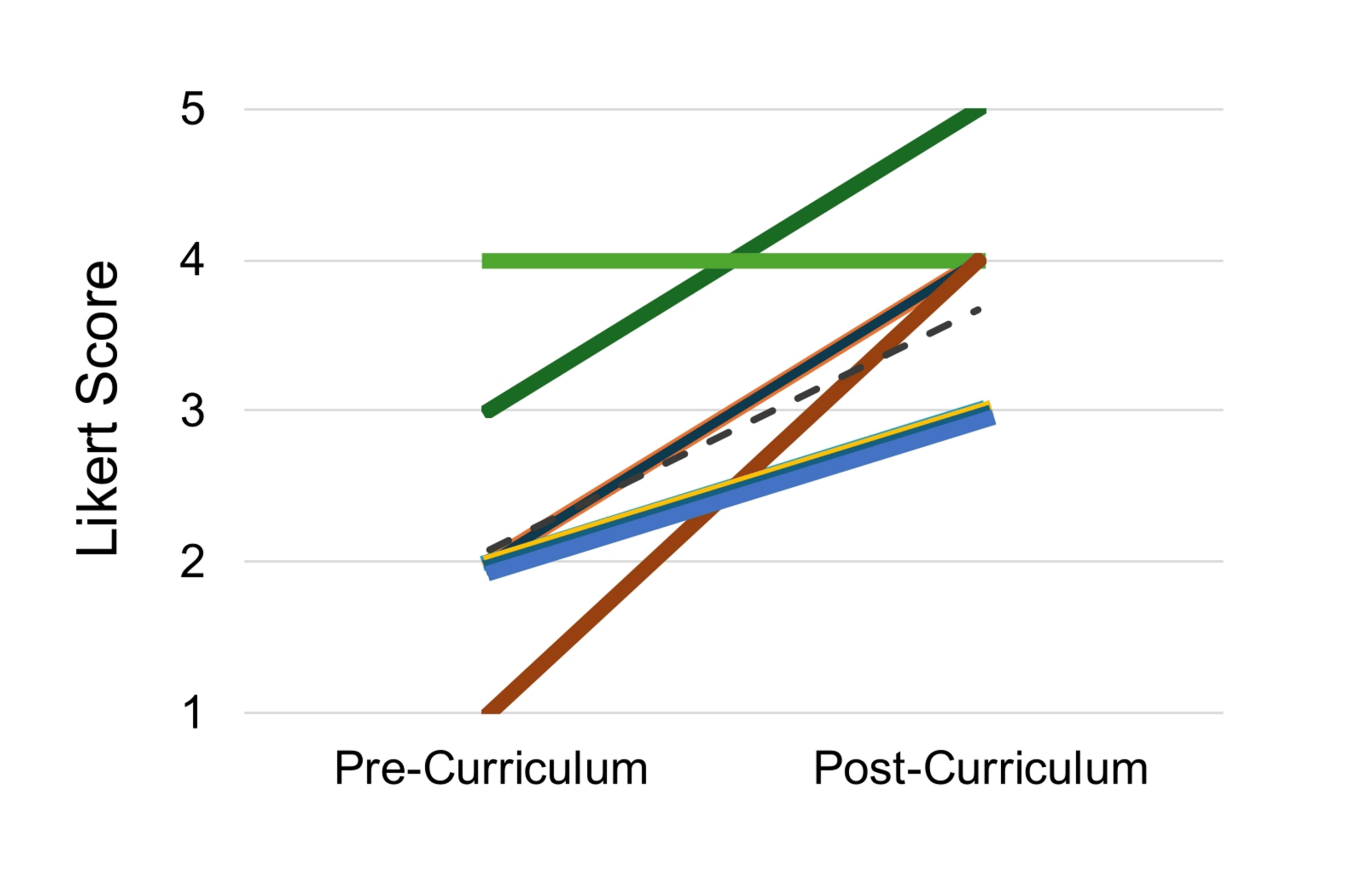
Participant Preparedness to Engage in Future QI Efforts Paired Likert score describing participant self-reported preparedness to engage in future quality improvement (QI) efforts, significantly increased from an average Likert score 2.1 pre-curriculum to 3.7 after the curriculum, p<0.01. Each color represents a different individual, N=8 for paired scores due to not all participants completing both Likert surveys pre-and post-implementation of curriculum.

The pilot PSQI curriculum was developed and successfully piloted during the 2023-2024 academic year within a two-month period of resident didactics. In total, 13 residents were able to participate, however, eight (62%) residents completed both pre- and post-curriculum surveys and in-training exams. On the five-point Likert survey (one = not at all, five = extremely), participant's self-reported preparedness to engage in quality improvement efforts in the future significantly increased with the average Likert score increasing from 2.1 (slightly prepared) pre-curriculum to 3.7 (moderately-very prepared) after the curriculum, p<0.01 (Figure 1).

**Figure 2:**
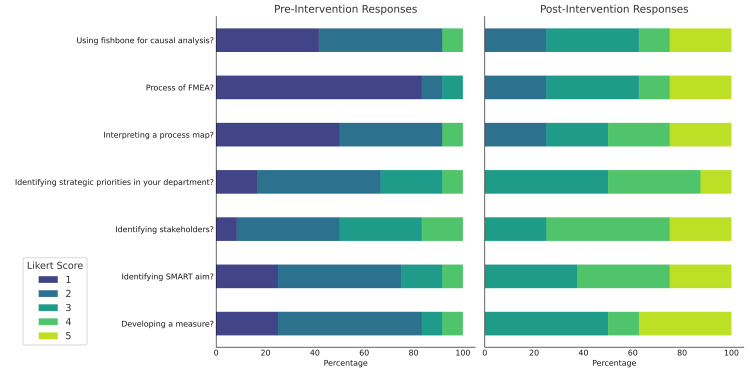
Participant Comfort Level With PSQI tools Average Likert scores pre- and post-curriculum evaluating different patient safety and quality improvement (PSQI) tools reviewed and simulated during the curriculum. N=8 FMEA: Failure Modes and Effects Analysis, SMART: specific, measurable, achievable, relevant, time-bound

In addition, Likert scores demonstrated significantly improved comfort with utilizing PSQI tools including identifying departmental strategic priorities; identifying stakeholders; identifying a SMART aim; developing a measure; interpreting a process; developing a fishbone diagram; and understanding the Failure Modes and Effects Analysis (FMEA) process, p<0.01 (Figure 2).

**Figure 3:**
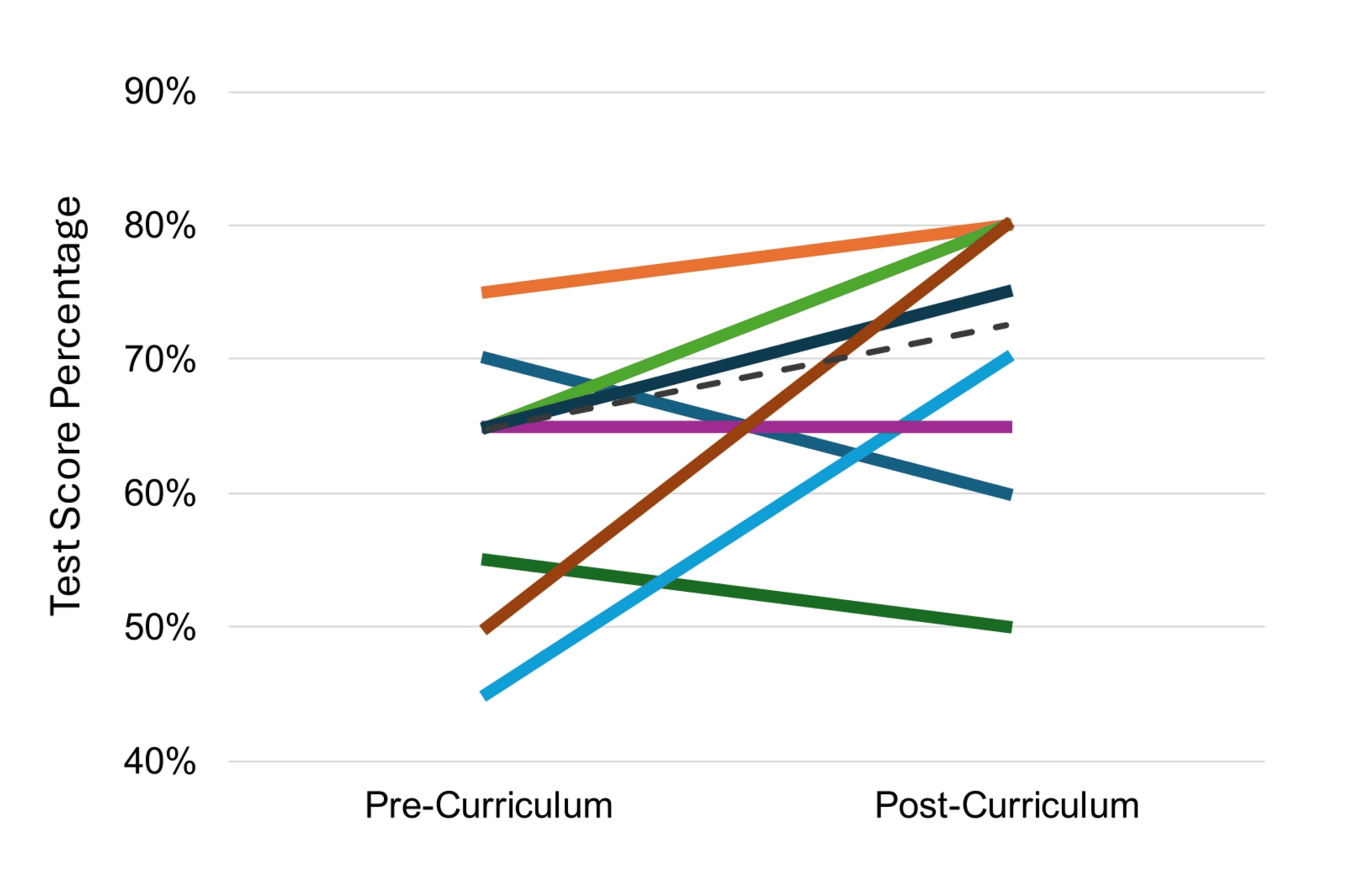
Participant PSQI in-training exam scores Paired participant patient safety and quality improvement (PSQI) in-training exam scores. The average test score improved from 57% to 70%, p<0.01. Each color represents a different individual, N=8 for paired scores due to not all participants completing both in-training exams pre- and post-implementation of curriculum.

Participants also had increased interest in leading future QI efforts after the curriculum with Likert scores rising from 2.8 to 3.4, although data was not statistically significant. Evaluation of paired exam scores demonstrated that the curriculum significantly improved participant understanding of key PSQI tools and radiation safety. The average test score improved from 57% to 70%, p<0.01 (Figure 3).

In the qualitative discussion, the overwhelming majority of trainees felt that patient safety and quality would be important to their future practice, 92% (12/13). Prior to the curriculum, residents were most interested in learning about specific tools for developing and evaluating a patient safety or QI project such as “How to make SMART aims”, “How to develop a measure”, “Understanding the difference between a near miss and a misadministration”. At the end of the pilot curriculum, resident interests targeted the next steps towards implementing a QI project including “I need to learn the department more to better identify areas of QI project targeting”, and “I have a better understanding of how FMEA is made.” These responses demonstrated the participants acknowledge the value of PSQI and themes from post-curriculum evaluations suggested increased interest in development of a QI project and patient safety analyses such as FMEA.

## Discussion

The implementation of this PSQI pilot curriculum and simulated QI project resulted in improved trainee understanding of QI techniques, development of skills to effectively engage in QI projects and increased interest in leading future QI projects.

Our curriculum built upon several mechanisms identified in previous quality improvement curricula including improving resident engagement by using simulation-based learning [16]. At the start of the educational sessions, we discussed the expectation of the residents working as a team to complete a portion of the simulated QI project with each didactic session. The curriculum enabled residents to develop and complete an iteration of a QI project without common barriers such as lack of supportive leadership or lack of timely or relevant data [17]. By incorporating radiation oncology-specific examples and practice-based learning, trainees gained a deeper comprehension of how PSQI principles and methodologies can be applied to their clinical practice. Their post-implementation surveys also demonstrate participants have a significantly increased interest in leading QI projects and contributes to the culture of safety within the radiation oncology department. This curriculum also successfully tailored education to board examination preparation and reinforced important patient safety terminology and radiation treatment plan and delivery quality assurance with improvement in mean PSQI in-training exam scores. By implementing this curriculum during structured and protected didactic time, the residents were able to operate as one team and have a mentored experience as well as dedicated time to learn, which has been identified as one of the greatest barriers to incorporating QI in residency education [18].

The limitation of our curriculum includes the small pilot cohort of residents and need to replicate the curriculum in future years. Although all residents are expected to attend didactic sessions, elective clinical rotations or medical leave meant that some residents missed lectures and not all resident data was captured using the survey and in-training exam scores, overall decreasing the strength of the data. Additionally, the broader impact of this curriculum including the evaluation of how many residents successfully undertake a PSQI project in residency or in their future clinical practice during maintenance of certification is another measure that has not been assessed and requires greater longitudinal follow-up. It is notable that while Likert scores for preparedness to engage in future QI efforts, comfort level with PSQI tools, and test scores improved after the intervention, overall scores remained significantly lower than their maximum potential. Likely repeated exposure to this PSQI curriculum throughout the four years of training is more likely to improve knowledge and skills in PSQI as well application towards individual PSQI initiatives.

Radiation oncology programs are inherently small and residents work with attendings in a one-on-one apprenticeship model. This leads to difficulty accessing faculty QI experts for residents to develop and lead their own QI projects based on clinical interest. Given the identified lack of qualified faculty with expertise in PSQI [19], future iterations of this project could include interdepartmental collaborations or multi-institutional curricula accessible through an online platform. A future objective of this curriculum will be the development of a PSQI curriculum framework that can be disseminated to radiation oncology residency programs throughout the country with a simulated QI project based on institutional priorities. The development of this educational series can be discussed through webinars or podcast formats online to assist faculty with training or experience in QI in the mentorship of other clinician educators. It is important to foster QI skill development in interested faculty who can identify opportunities for residents to participate in institutional QI efforts, which is a key strategy in implementing QI curriculum to meet ACGME common requirements [20]. This interdisciplinary engagement will enhance their understanding of the importance of teamwork, effective communication, and collaborative problem-solving in QI projects. Faculty career development in PSQI is often limited during their clinical practice and methods to increase participation include implementation of faculty development initiatives, acknowledge of awards, grant funding and providing continued medical education courses and credit for participation [19].

## Conclusions

Our PSQI curriculum and simulated QI project workshop demonstrated improved qualitative and quantitative PSQI knowledge base and increased interest in pursuing PSQI in participants. This foundational PSQI curriculum can effectively equip physicians with the skills and knowledge needed to deliver high-quality, safe, and patient-centered care. Residents benefit from continuing exposure and opportunities to get involved in institutional QI efforts to help improve buy-in and practical application of QI concepts in their clinical practice.

## Appendices

Pre and Post-Implementation Survey. Likert Score [1 (Not at all)-5 (Extremely)]

How prepared do you feel to engage in QI efforts in the future?

Do you think interprofessional collaborative practice is important to improvement health care quality?

Do you have interest in leading QI efforts in your future career?

What is your comfort level with the following QI skills:

Identifying strategic priorities in your department?

Identifying stakeholders?

Identifying SMART aim?

Developing a measure?

Interpreting a process map?

Using fishbone for causal analysis?

Process of FMEA?

Pre-Implementation open ended question:

What are some topics in patient safety and quality improvement that you want to learn from these didactics?

Will patient safety and quality metrics be important to your future practice as a physician?

Post-Implementation open ended question:

What are some topics in patient safety and quality improvement that you have learn from these didactics?

Will patient safety and quality metrics be important to your future practice as a physician?
